# Supplementary material for: A Cellular Automaton Simulation for Predicting Phase Evolution in Solid-State Reactions
Source: Chem Mater. 2024 Dec 18;37(1):210–23. doi: 10.1021/acs.chemmater.4c02301 (PMC11736680; doi:10.1021/acs.chemmater.4c02301)
Supplement: Supplementary file 2 — cm4c02301_si_002.pdf [file cm4c02301_si_002.pdf]

Supporting Information: A Cellular Automaton Simulation for Predicting Phase Evolution in Solid-State Reactions

Max C. Gallant,[†,‡] Matthew J. McDermott,[†,‡] Bryant Li,[†,‡] and Kristin A. Persson[*,†,‡]

†*Materials Sciences Division, Lawrence Berkeley National Laboratory, Berkeley, California, 94720, U.S.A.*

‡*Department of Materials Science and Engineering, University of California, Berkeley, California, 94720, U.S.A.*

E-mail: kapersson@lbl.gov

Swapping probability as a function of temperature

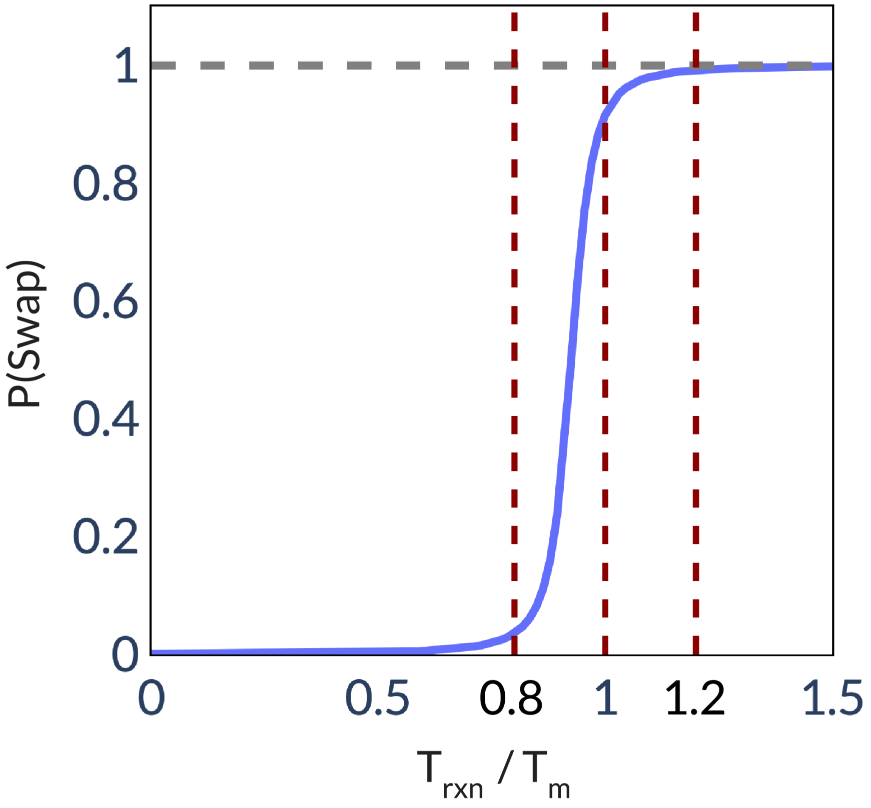

Figure S1: Swapping probability during the Melt Swap action The probability of neighboring cells swapping during an application of the evolution rule is a function of the ratio of the melting point of the phase occupying the evolving cell and the current reaction temperature. The onset of swapping behavior occurs at 80% of the melting temperature of the phase in the evolving cell, and reaches 100% by the time the reaction temperature exceeds this melting point by 20%.

Description of the Reaction Progression action

The Reaction Progression evolution rule action is designed such that mass is conserved when it is applied repeatedly. This conservation is achieved by the sufficient sampling of probability distributions that are constructed based on the stoichiometry of the reaction. An example presented here illustrates this process.

Consider two neighboring cells, denoted C_1 and C_2, the contents of which are undergoing a reaction. C_1 is occupied by phase A and C_2 is occupied by phase B. Assume the following reaction (whose coefficients are expressed in units of volume) has been selected to proceed between these cells:

$$A + 2B \rightarrow 3C + 4D \tag{S2}$$

After the reaction is selected to proceed, the new state of each of these two cells is determined independently.

Whether or not the contents of each cell are consumed is determined by treating the coefficients of the reactants in (S2) as frequencies. In other words, when this reaction proceeds, it consumes A one third of the time and B two thirds of the time. To capture this, we begin by constructing a distribution over the reactants using these coefficients as weights. Formally, for reaction T with reactants R and products P and stoichiometric coefficients r and p, the probability that reactant r_i is consumed is given by the following:

$$P(R_i \text{ is consumed}) = \frac{r_i}{\sum_{j \in r} r_j} \tag{S3}$$

$$P(R_i \text{ is not consumed}) = \frac{\sum_{j \in r, j \neq i} r_j}{\sum_{j \in r} r_j} \tag{S4}$$

Applying this to the reaction in (S2), the following probabilities are generated for C_1:

$$P(\text{A is consumed}) = \frac{1}{1+2} = \frac{1}{3} \qquad P(\text{A is not consumed}) = \frac{2}{1+2} = \frac{2}{3}$$

And the following are generated for C_2:

$$P(\text{B is consumed}) = \frac{2}{1+2} = \frac{2}{3} \qquad P(\text{B is not consumed}) = \frac{1}{1+2} = \frac{1}{3}$$

For each cell, a random draw from the corresponding distribution is performed to determine whether or not the contents are consumed. As illustrated above, there is a 1/3 chance that C_1 (which contains phase A) is consumed and a 2/3 chance that it is not. Similarly, there is a 2/3 chance that C_2 (which contains phase B) is consumed, and a 1/3 chance that it is not. These draws are performed independently.

Next, for each of the cells that is consumed (it might have been one of them, both of them, or neither of them), a product phase is selected. To make this selection we again treat the reaction coefficients as frequencies. Again considering reaction T involving reactants R (with coefficients r) and products P (with coefficients p), the probability of producing of producing phase P_i is given as follows:

$$P(P_i \text{ is produced}) = \frac{p_i}{\sum_{j \in p} p_j} \tag{S5}$$

For the reaction in (S2), these probabilities work out to be:

$$P(\text{C is produced}) = \frac{3}{3+4} = \frac{3}{7} \qquad P(\text{D is produced}) = \frac{4}{3+4} = \frac{4}{7}$$

Using these likelihoods, at each consumed cell, a random draw is performed, and the content of the cell is replaced by the phase resulting from the draw.

It is important to note that reactions do not conserve volume. In this example, the reaction consumes 3 units of volume and produces 7 units of volume. To capture this, the volume of each consumed cell is scaled by this ratio:

$$V_{new} = \frac{V_{products}}{V_{reactants}} * V_{old} \tag{S6}$$

Finally, the simulation state is updated with the new volume and product phase for each cell which was consumed by the reaction (if any), and the next application of the evolution rule begins.

Outcome convergence

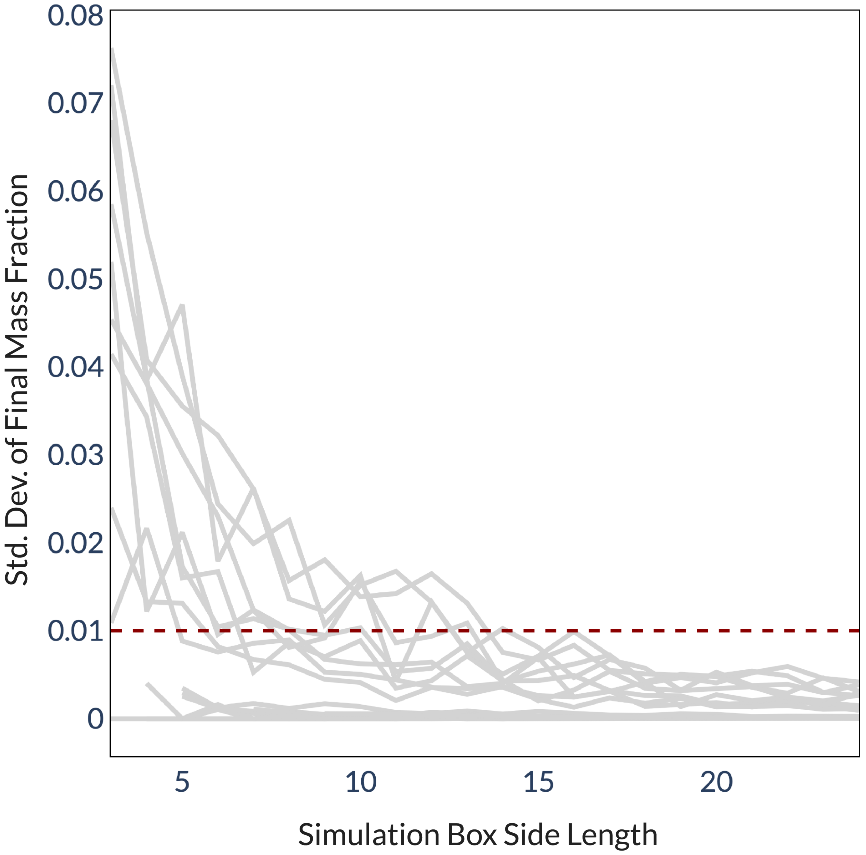

Figure S2: Convergence of the standard deviation of final mass fraction for each phase For each in a range of simulation sizes (side lengths between 3 and 24), 10 simulations were run. For each phase that appeared, the mass fraction attained at the end of each simulation was calculated. For each simulation size, the standard deviation of these final mass fraction values was calculated on a per phase basis. The plot above shows the convergence behavior of these standard deviations on a per phase basis (one trace per phase). Notably, for simulation boxes with side length greater than 15 cells, the standard deviation of the final mass fraction of each phase over repeated simulations is less than 1%.

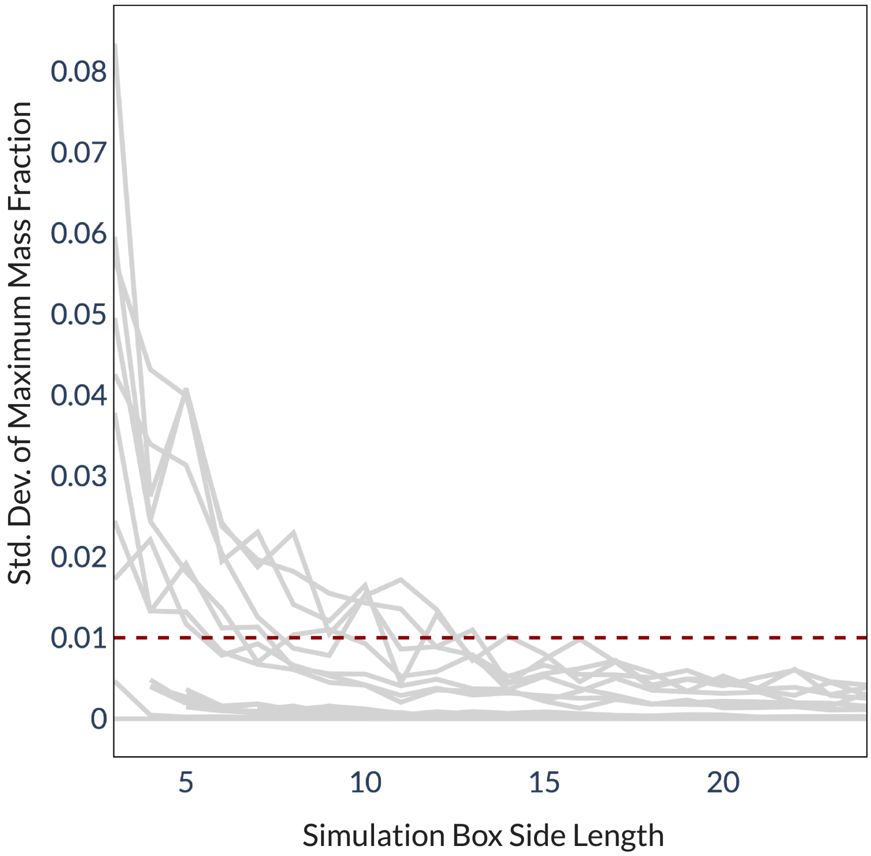

Figure S3: Convergence of the standard deviation of maximum mass fraction achieved for each phase For each in a range of simulation sizes (side lengths between 3 and 24), 10 simulations were run. For each phase that appeared, the maximum mass fraction attained at any point during each simulation was calculated. For each simulation size, the standard deviation of these maximum mass fraction values was calculated on a per phase basis. The plot above shows the convergence behavior of these standard deviations on a per phase basis (one trace per phase). Notably, for simulation boxes with side length greater than 15 cells, the standard deviation of the maximum mass fraction of each phase over repeated simulations is less than 1%.

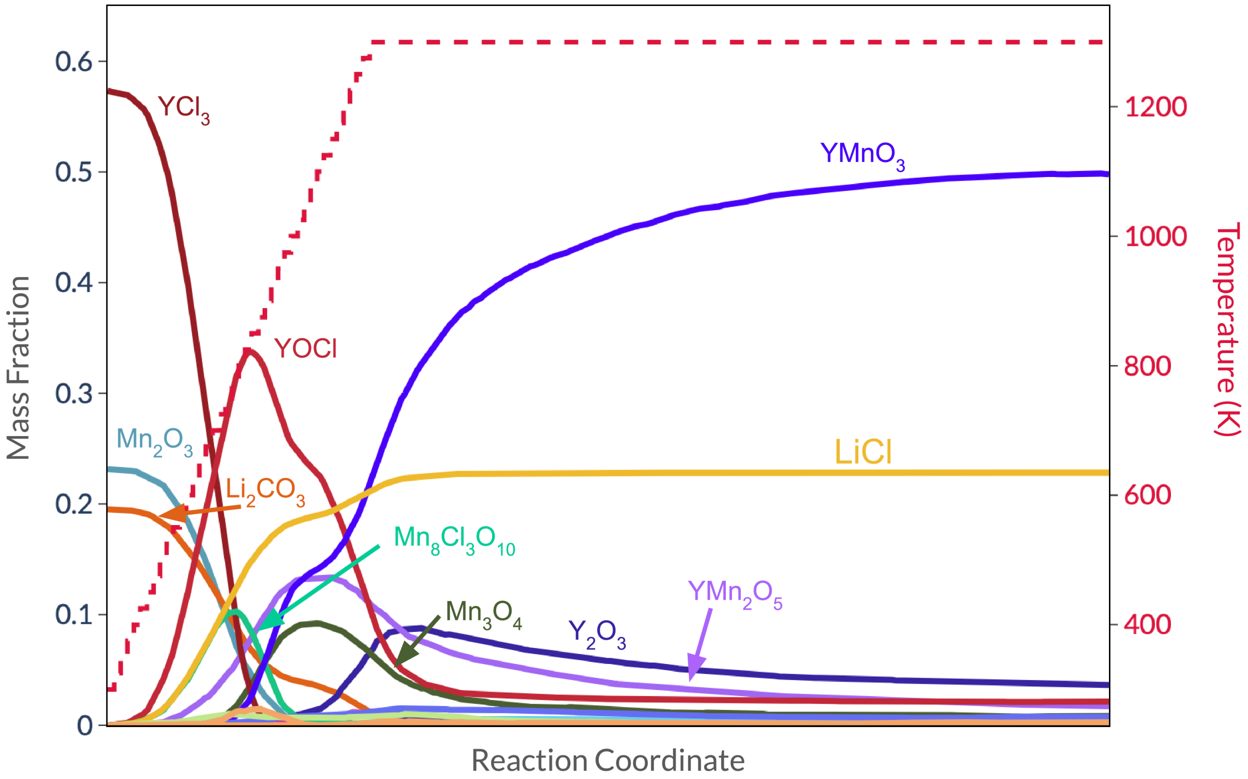

Figure S4: Extended trajectory for $YMnO_3$ reaction A longer trajectory for the $YMnO_3$ reaction showing the plateauing of each of the eventual products.

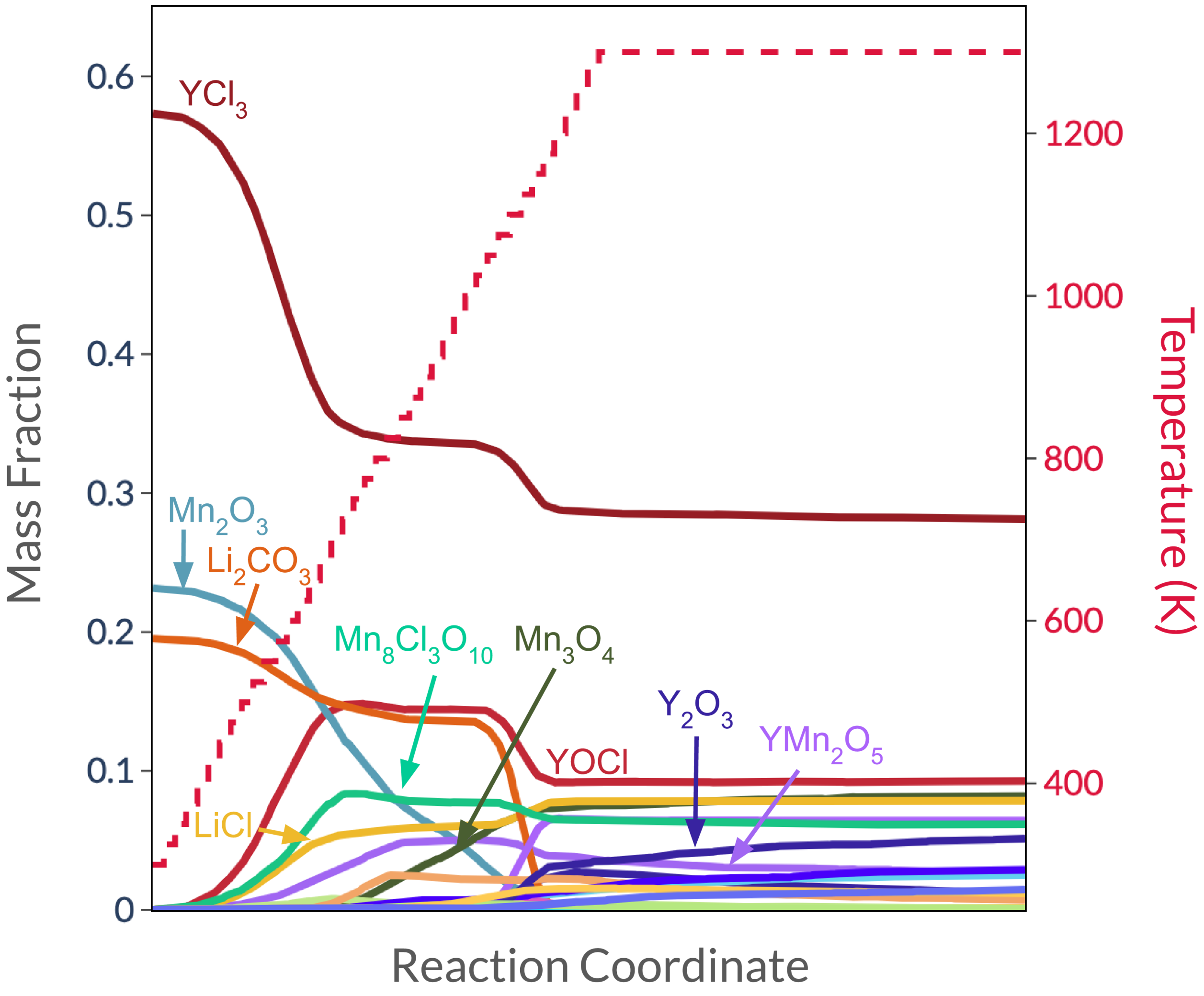

Figure S5: Simulation results for $YMnO_3$ using an alternative evolution rule with no melt representation. The result shown here was produced using a simulation that excludes the Melt-Swap action from the evolution rule. This illustrates the significant improvement that this part of the evolution rule facilitates. In this result, the reaction stalls at an intermediate point because the reactant particles are not sufficiently mixed (an effect achieved by the presence of a melt, which, in a real solid-state reaction, significantly improves transport.

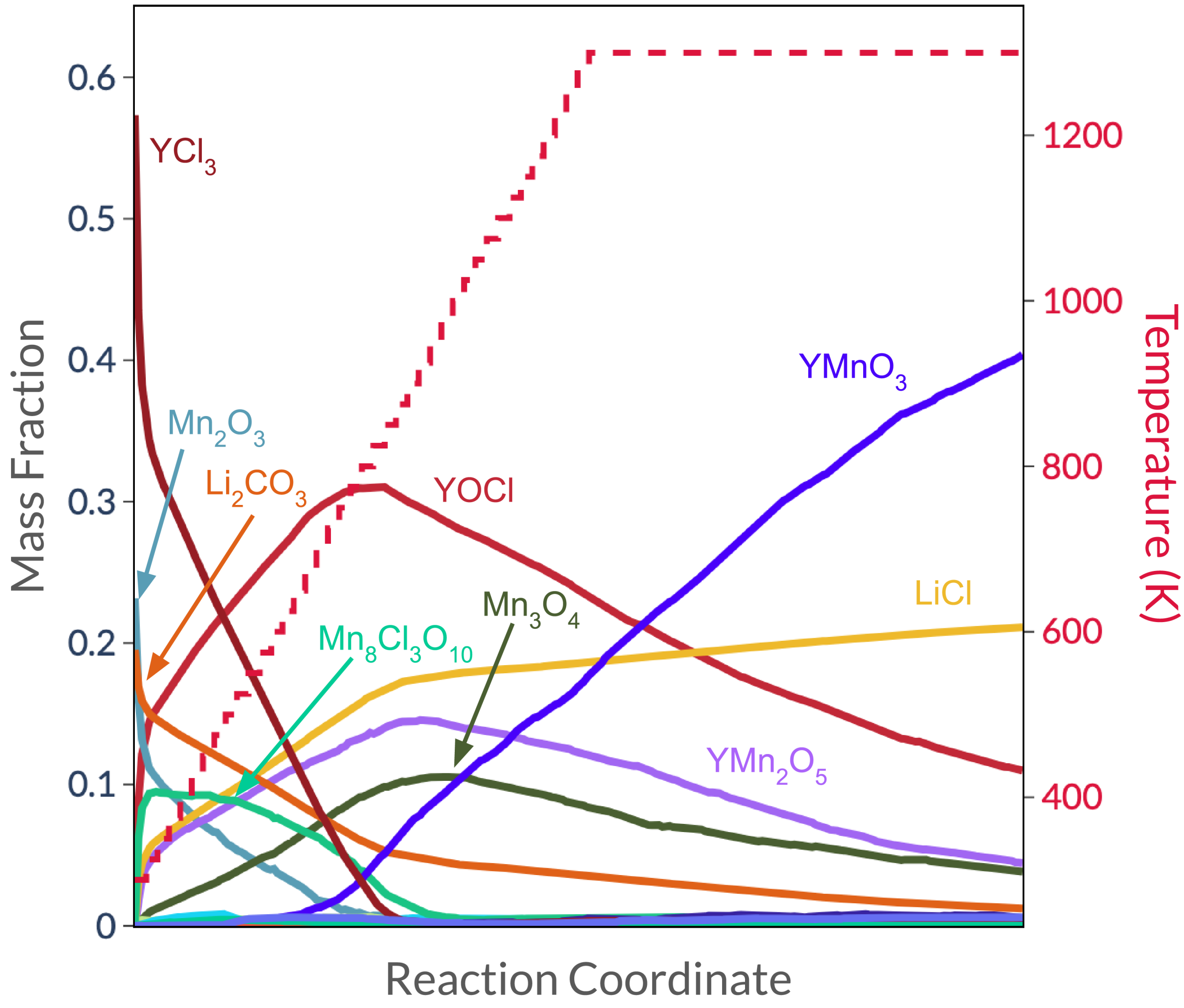

Figure S6: Simulation results for $YMnO_3$ using an alternative score function that excludes Tamman's Rule and utilizes only thermodynamics. The result shown here was produced using a simulation that excludes the Tamman's Rule heuristic from score function. This figure illustrates the two main enhancements that the Tamman heuristic provides to the simulation. Specifically, without the Tamman's Rule heuristic, 1) reactions occur with speed at even the lowest temperature - a completely nonphysical result, and 2) the lower reactivity of hard (higher T_m) materials is not distinguished. In this particular case study, this exclusion yields a different reaction pathway: the Y_2O_3 intermediate (prominent in the original result and in the experiment), achieves no notable prevalence.

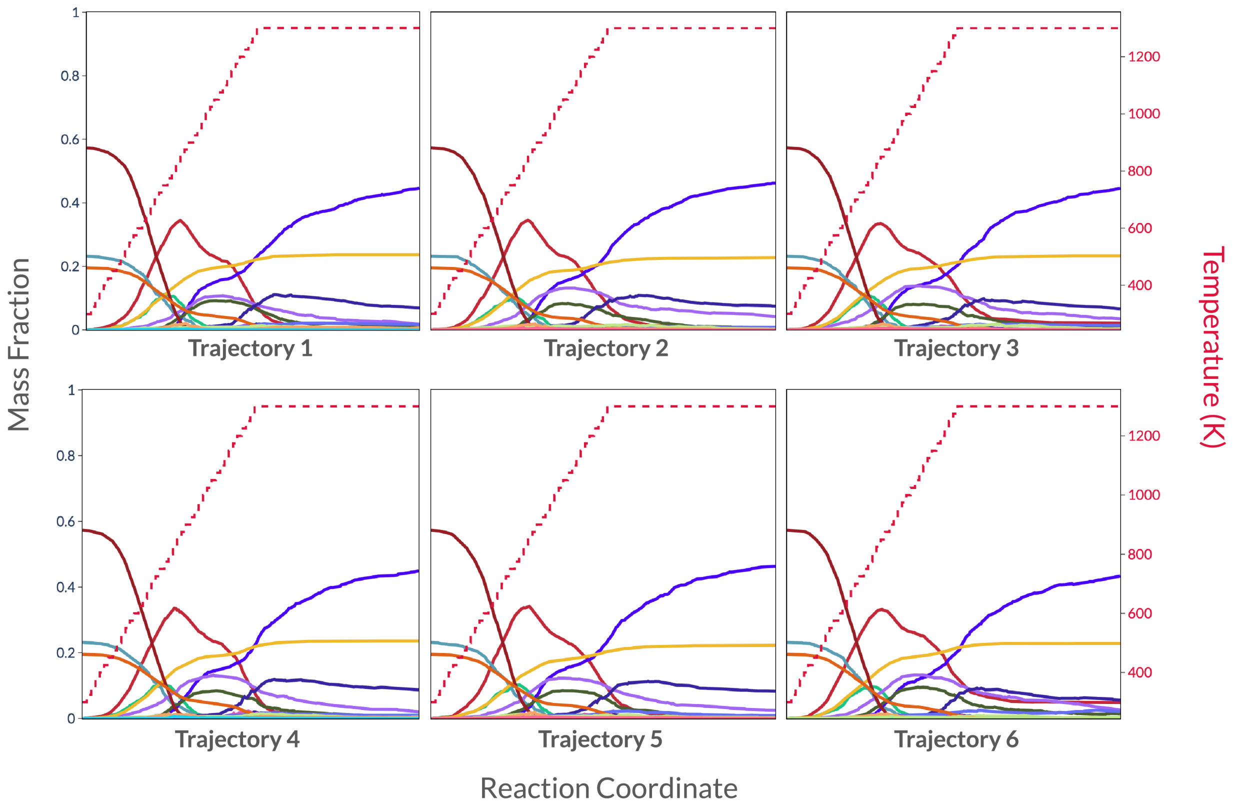

Figure S7: Illustration of the fluctuations between trajectories with different random initial configurations for a $YMnO_3$ reaction recipe. The six trajectories shown here were averaged together to produce the results presented in the main manuscript for the $YMnO_3$ synthesis reaction. While there are some differences between trajectories, the qualitative features of the result do not change significantly between them.
